# Analysis on artificial intelligence-based chest computed tomography in multidisciplinary treatment models for discriminating benign and malignant pulmonary nodules

**DOI:** 10.1016/j.clinsp.2025.100734

**Published:** 2025-08-05

**Authors:** Xian-Yan Liu, Fa-Cheng Shan, Hui Li, Jian-Bo Zhu

**Affiliations:** a Department of Respiratory Medicine, Binzhou People's Hospital, Binzhou, Shandong, China; b Department of Radiotherapy, Binzhou People's Hospital, Binzhou, Shandong, China

**Keywords:** MDT, Pulmonary nodules, Chest scan, Artificial intelligence, Combined diagnostic

## Abstract

**Objective:**

To evaluate the effectiveness of AI-based chest Computed Tomography (CT) in a Multidisciplinary Diagnosis and Treatment (MDT) model for differentiating benign and malignant pulmonary nodules.

**Methods:**

This retrospective study screened a total of 87 patients with pulmonary nodules who were treated between January 2019 and December 2020 at Binzhou People's Hospital, Qingdao Municipal Hospital, and Laiwu People's Hospital. AI analysis, MDT consultation, and a combined diagnostic approach were assessed using postoperative pathology as the reference standard.

**Results:**

Among 87 nodules, 69 (79.31 %) were malignant, and 18 (20.69 %) were benign. AI analysis showed moderate agreement with pathology (κ = 0.637, *p* < 0.05), while MDT and the combined approach demonstrated higher consistency (κ = 0.847, 0.888, *p* < 0.05). Sensitivity and specificity were as follows: AI (89.86 %, 77.78 %, AUC = 0.838), MDT (100 %, 77.78 %, AUC = 0.889), and the combined approach (100 %, 83.33 %, AUC = 0.917). The accuracy of the combined method (96.55 %) was superior to MDT (95.40 %) and AI alone (87.36 %) (*p* < 0.05).

**Conclusion:**

AI-based chest CT combined with MDT may improve diagnostic accuracy and shows potential for broader clinical application.

## Highlights

• Artificial intelligence based chest Computed Tomography (CT) is a diagnosis.
• Eighty-seven patients with pulmonary nodules who underwent chest CT scanning.
• Analysis shows 69 malignant (79.3 %) and 18 benign-nodules (20.7 %) with 87 patients.
• Accuracy of combined diagnostic method is 96.55 % in discriminating these nodules.
• Compared to AI, AI-based chest CT combined with the MDT model had greater value.

## Introduction

Lung cancer remains one of the most prevalent malignancies worldwide, with a high annual incidence. Early symptoms are often subtle, such as paroxysmal dry cough with minimal sputum, while disease progression may lead to vague or dull chest pain. As a result, many patients are diagnosed at advanced stages, missing the optimal treatment window and facing poor prognoses.^1^^,^^2^ Pulmonary nodules are a common radiological manifestation of lung cancer. They typically exhibit diverse morphologies and lack specific clinical signs, making accurate diagnosis difficult. Advances in Computed Tomography (CT) are leading to an increased detection rate of pulmonary nodules, a heavier workload for radiologists, and greater risk of diagnostic errors. Traditional diagnostic approaches, largely dependent on physician experience, are susceptible to missed diagnoses and misdiagnosis,^3^ which can delay treatment, burden patients psychologically, and waste medical resources. Accurate differentiation between benign and malignant pulmonary nodules is therefore essential.

Artificial Intelligence (AI) has shown great promise in medical imaging by simulating and extending human cognitive abilities. Applied to chest CT, AI can assist in image acquisition, nodule detection and segmentation, feature extraction, and malignancy prediction through high-throughput processing.^4^ It is especially effective in identifying new nodules, monitoring changes in size or density, and improving diagnostic efficiency compared to manual interpretation.^5^^,^^6^ Nevertheless, AI remains an auxiliary tool, and final diagnosis must be confirmed by medical professionals.

In recent years, the Multidisciplinary Diagnosis and Treatment (MDT) model has played a vital role in standardizing lung cancer diagnosis and treatment, particularly in regions where healthcare resources are unevenly distributed.^7^ Integrating AI into MDT workflows may enhance diagnostic accuracy and improve efficiency. For example, a European multicenter study reported that a Convolutional Neural Network (CNN) ‒ based AI model, when combined with MDT consultation, achieved an Area Under the Curve (AUC) of 0.91 ‒ superior to either method alone. Kheir et al. further pointed out that AI-MDT collaboration could reduce the misdiagnosis rate of lung nodules to below 5 %.^8^ Similarly, a study by Fan et al. in China showed that combining AI with a multidisciplinary approach improved diagnostic sensitivity to 97 %.^9^ These international advances not only highlight the clinical potential of the AI-MDT model, but also provide an important reference for the cross-center collaborative design of this study.

Therefore, the present study aimed to evaluate, in a multicenter context, the diagnostic performance of AI-based chest CT combined with the MDT model in distinguishing benign from malignant pulmonary nodules, with the goal of supporting further refinement and validation of this integrated approach.

## Materials and methods

This retrospective study screened a total of 87 patients with pulmonary nodules who were treated between January 2019 and December 2020 at Binzhou People's Hospital, Qingdao Municipal Hospital, and Laiwu People's Hospital. The cohort comprised 40 males and 47 females, aged between 21- and 73-years, with a mean age of 50.64±8.32 years. The Body Mass Index (BMI) ranged from 19.0 to 28.0 kg/m^2^, with a mean BMI of 23.76±2.04 kg/m^2^. The diameters of the pulmonary nodules varied from 3 mm to 30 mm, with an average diameter of 9.58±3.67 mm. This study was approved by the Ethics Committee of Binzhou People's Hospital (Approval n° LL-2023–008).

### Inclusion and exclusion criteria

Inclusion criteria were as follows: 1) Pulmonary nodules highly suspected to be malignant, indeterminate for malignancy, or causing severe patient anxiety that significantly interfered with daily life and required urgent surgical intervention; 2) Patients who underwent surgical resection of pulmonary nodules followed by histopathological examination with confirmed pathological diagnoses; 3) Patients who received CT-based AI analysis prior to surgery; and 4) Patients and their family members who were fully informed about the study and provided signed informed consent.

Exclusion criteria were as follows: 1) History of a confirmed malignant tumor; 2) Pulmonary nodules larger than 3 cm in diameter or accompanied by obvious pleural effusion, atelectasis, or hilar lymphadenopathy; 3) Incomplete chest CT imaging data or missing MDT diagnostic records; and 4) Contraindications to surgery, poor overall health status, intolerance to surgical treatment, or conditionslikely to adversely affect postoperative quality of life.

### Chest computed tomography

Chest imaging was performed using either a Philips MX 16 EVO spiral CT scanner or a Siemens SOMATOM Definition AS+ 128-row helical CT scanner. The scan range extended from the lung apex to the base, with patients instructed to hold their breath after ifollowing inhalation throughout the scan. The scan parameters were as follows: tube voltage = 120 kV, tube current = 200 mA, pitch = 1.375:1, slice thickness = 5 mm, matrix = 512 × 512, and reconstructed slice thickness = 1.5 mm. After acquisition, the original images were reconstructed as single-energy images.

### AI analysis and diagnostic methods

In this study, a pulmonary nodule detection AI system developed by Tianchi Medical Company was utilized for chest CT image analysis. Chest CT images were uploaded to the AI platform, which enabled automated detection and accurate visualization of pulmonary nodules. The system provided detailed information regarding each nodule, including its size, location, density, and volume.

The AI algorithm estimated the probability of malignancy for each detected nodule. Following the diagnostic threshold criteria reported by Du et al.,^10^ nodules with a predicted malignancy probability >70 % were considered malignant in this study.

The underlying AI model was constructed using a deep Convolutional Neural Network (CNN) architecture. It was trained on a dataset of over 1000 annotated chest CT scans, which included both benign and malignant pulmonary nodules confirmed through histopathological examination or long-term clinical follow-up. All annotations were performed by experienced radiologists.

### Diagnostic methods for MDTs

An MDT team comprising specialists from the Thoracic Surgery, Radiology, Oncology, and Respiratory Departments of Binzhou People’s Hospital, Qingdao Municipal Hospital, and Laiwu People’s Hospital was formed. Each MDT consultation session involved at least five senior-level physicians, including at least one thoracic surgeon, one radiologist, one respiratory physician, and one oncologist. MDT consultations were conducted once weekly at the Multidisciplinary Consultation Center, and additional ad hoc meetings were held for urgent cases when necessary.

During the decision-making process, all MDT experts were blinded to the AI system’s malignancy probability scores and outputs to avoid potential bias. The MDT decisions were based solely on clinical presentation, imaging interpretation, and professional experience. If two or more experts reached a unanimous agreement on the need for surgical intervention, the patient was referred for inpatient surgery. In cases of disagreement or diagnostic uncertainty, the patient was advised to undergo follow-up imaging and observation.

### Observation indicators

1) Postoperative pathological diagnoses were recorded. 2) Using the postoperative pathological results as the reference standard, the diagnostic performance of AI analysis, MDT consultation, and the combined diagnostic approach was evaluated for distinguishing between and malignant pulmonary nodules.

### Statistical analysis

The data were processed using SPSS 23.0 software. Measurement data were expressed as mean ± standard deviation ($\bar{x}$±*s*) and compared using the *t*-test, while enumeration data were expressed as n ( %) and compared using the χ^2^ test. The kappa statistic was used to assess the consistency between AI analysis, MDT consultation, and the combined diagnostic method in distinguishing benign from malignant pulmonary nodules compared to pathological diagnostic results, with 0.4 < Kappa value < 0.75 indicating good consistency and Kappa value ≥ 0.75 indicating excellent consistency. The predictive performance was analyzed by plotting Receiver Operating Characteristic (ROC) curves. Sensitivity, specificity, and accuracy were reported along with their 95 % Confidence Intervals, which were calculated using the Wilson score method. A p-value < 0.05 was considered to indicate statistical significance. Imaging protocols at all three participating centers were harmonized to ensure consistency.

## Results

### Postoperative pathological diagnosis results in patients with pulmonary nodules

Postoperative pathological analysis showed that among the 87 patients with pulmonary nodules, 69 (79.31 %) had malignant lesions, including 65 cases of adenocarcinoma, 1 case of small cell lung cancer, 1 case of squamous cell carcinoma, and 2 cases of lymphoma. The remaining 18 patients (20.69 %) had benign nodules, comprising 5 cases of atypical adenomatous hyperplasia, 8 cases of inflammatory granuloma, 2 cases of inflammatory pseudotumor, 2 cases of fibrous tissue, and 1 case of a benign pulmonary nodule.

### Diagnostic efficacy of AI analysis, MDT consultation, and the combined diagnostic method in differentiating benign and malignant pulmonary nodules

The diagnostic performance of the AI analysis in differentiating benign from malignant pulmonary nodules showed substantial agreement with the postoperative pathological findings (κ = 0.637, *p* < 0.05). In contrast, the MDT consultation and the combined diagnostic approach demonstrated excellent concordance with pathological results (κ = 0.847 and 0.888; *p* < 0.05). Both MDT consultation and the combined diagnostic approach exhibited significantly higher sensitivity in identifying benign pulmonary nodules compared to AI analysis (*p* < 0.05). Moreover, the combined method achieved superior diagnostic accuracy over AI analysis alone (*p* < 0.05). The sensitivity, specificity, and accuracy of each diagnostic method are summarized in Table 4, with 95 % Confidence Intervals provided. For additional details, refer to Table 1, Table 2, Table 3, Table 4.

**Table 1:** Diagnostic efficacy of AI analysis in differentiating benign and malignant pulmonary nodules (n).

AI analysis	Pathological findings		Total
	Malignant nodule	Benign nodule	
Malignant nodule	62	4	66
Benign nodule	7	14	21
Total	69	18	87

AI, Artificial Intelligence.

**Table 2:** Diagnostic efficacy of MDT consultation in identifying benign and malignant pulmonary nodules (n).

MDT consultation	Pathological findings		Total
	Malignant nodule	Benign nodule	
Malignant nodule	69	4	73
Benign nodule	0	14	14
Total	69	18	87

MDT, Multidisciplinary Diagnosis and Treatment.

**Table 3:** Diagnostic efficacy of the combined diagnostic method in differentiating benign and malignant pulmonary nodules (n).

The combined diagnostic method	Pathological findings		Total
	Malignant nodule	Benign nodule	
Malignant nodule	69	3	72
Benign nodule	0	15	15
Total	69	18	87

**Table 4:** Comparison of the sensitivity, specificity, and accuracy of the three diagnostic methods ( %).

Group	Sensitivity (95 % CI)	Specificity (95 % CI)	Accuracy (95 % CI)
AI analysis	89.86 (62/69)	77.78 (14/18)	87.36 (76/87)
	(80.25 %–95.78 %)	(52.36 %–93.59 %)	(78.68 %–93.53 %)
MDT consultation	100.00 (69/69)^a^	77.78 (14/18)	95.40 (83/87)
	(94.73 %–100.00 %)	(52.36 %–93.59 %)	(88.08 %–98.94 %)
The combined diagnostic method	100.00 (69/69)^a^	83.33 (15/18)	96.55 (84/87)^a^
	(94.73 %–100.00 %)	(58.58 %–96.42 %)	(89.67 %–99.33 %)
*χ*^2^	15.877	0.234	6.406
p	<0.001	0.889	0.041

Compared with the AI analysis.

a *p* < 0.05. AI, Artificial Intelligence; MDT, Multidisciplinary Diagnosis and Treatment; CI, Confidence Interval.

### ROC analysis of AI analysis, MDT consultation, and the combined diagnostic method for distinguishing benign and malignant pulmonary nodules

**Fig. 1:**
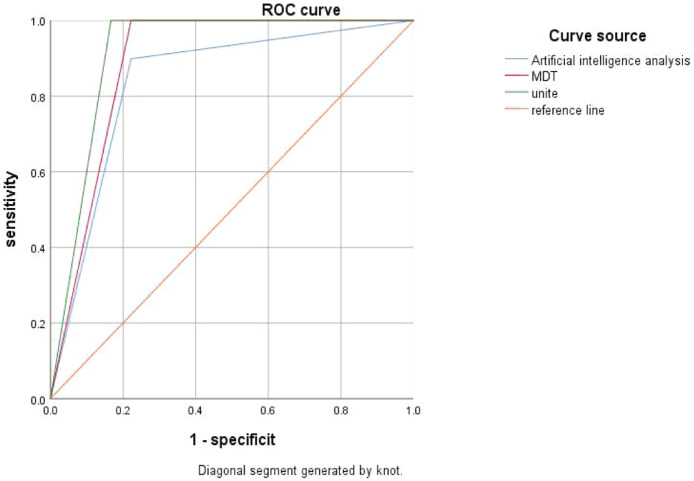
The ROC curves comparing the diagnostic performance of AI analysis, MDT consultation, and the unite AI-MDT method in differentiating benign from malignant pulmonary nodules. ROC, Receiver Operating Characteristic; AI, Artificial Intelligence; MDT, Multidisciplinary Diagnosis and Treatment.

The diagnostic outcomes of AI analysis, MDT consultation, and the combined diagnostic approach were treated as test variables, with the pathological nature of the pulmonary nodules defined as the state variable (1 = malignant nodule, 0 = benign nodule). The ROC curves were generated (Fig. 1). The results indicated that the AUC for distinguishing benign from malignant pulmonary nodules was 0.838 (95 % CI 0.718‒0.958) for AI analysis, 0.889 (95 % CI 0.773‒1.000) for MDT consultation and 0.917 (95 % CI 0.814‒1.000) for the combined diagnostic method (Table 5). A higher AUC value reflects superior diagnostic efficacy, and the AUC of the combined diagnostic approach was significantly higher than that of AI analysis alone (*p* < 0.05), suggesting that the combined approach can compensate for the limitations of AI in complex cases.

**Table 5:** ROC analysis of AI analysis, MDT consultation, and the combined diagnostic method for distinguishing benign and malignant pulmonary nodules.

Test variable	AUC	Standard error	p	95 % CI		Sensitivity	Specificity	Youden index
				Lower limit	Upper limit			
AI analysis	0.838	0.061	<0.001	0.718	0.958	0.899	0.778	0.677
MDT consultation	0.889	0.059	<0.001	0.773	1.000	1.000	0.778	0.778
The combined diagnostic method	0.917	0.052	<0.001	0.814	1.000	1.000	0.833	0.833

ROC, Receiver Operating Characteristic; AI, Artificial Intelligence; MDT, Multidisciplinary Diagnosis and Treatment; AUC, Area Under The Curve.

## Discussion

Lung cancer is characterized by high morbidity and mortality, underscoring the importance of early detection and treatment.^11^ Pulmonary nodules, defined as rounded or oval lesions ≤ 30 mm in diameter, are key targets for early diagnosis. Approximately 30 %‒40 % of such nodules are malignant, and timely surgical intervention can increase 5-year survival rates to over 90 %.^12^^,^^13^ However, delays in diagnosis significantly reduce survival, making accurate nodule assessment a clinical priority.

Dual-source low-dose spiral CT has enhanced pulmonary nodule detection but also increased the diagnostic burden. Differentiating benign from malignant nodules remains challenging. AI offers promising assistance by autonomously extracting imaging features, reducing reliance on subjective interpretation, and enhancing diagnostic consistency.^14^^,^^15^ Currently, AI has been widely applied in the diagnostic evaluation of pulmonary nodules, serving as an important auxiliary tool for clinicians in the diagnostic process. The diagnostic efficiency of AI has been widely recognized.^16^^,^^17^ In this study, postoperative pathological results were used as the gold standard, and the kappa value of the AI for distinguishing between benign and malignant pulmonary nodules was 0.637, indicating good consistency between the two methods. The sensitivity, specificity, and accuracy of the AI were 89.86 %, 77.78 %, and 87.36 %, respectively, with an AUC of 0.838, demonstrating its strong diagnostic value for benign and malignant pulmonary nodules. Previous studies have also linked features such as volume, longitudinal diameter, and CT values with malignancy probability.^18^^,^^19^

However, AI performance is limited by training data quality and representativeness. Sampling bias and uneven distribution of lesion types in training datasets can compromise model generalizability.^20^^,^^21^ The AI products used in this study were based on training data from specific medical centers, which may introduce selection bias due to geographic regions, population characteristics, or equipment differences. For example, the high percentage of malignant tumors in the training data (79.31 %) may have led to the model's lack of ability to recognize benign nodules.

The MDT model integrates expertise across specialties to improve diagnostic accuracy and standardize treatment planning.^22^ The results of this study showed that the consistency between the results of the MDT consultation for the identification of benign pulmonary nodules and the postoperative pathological diagnosis was excellent (κ = 0.847), with a diagnostic sensitivity, specificity, and accuracy of 100.00 %, 77.78 %, and 95.40 %, respectively, and the area under the AUC was 0.889, indicating that the MDT has high clinical utility in the differential diagnosis of benign and malignant pulmonary nodules. By bringing together diverse expertise, MDTs enhance diagnostic robustness, mitigate individual bias, and personalize treatment pathways. It can identify more suitable and authoritative treatment plans for patients and track and adjust them in real time so that some high-risk patients can receive personalized diagnosis and treatment, maximize patient safety, and avoid medical personnel being exposed to medical risks.^23^, ^24^, ^25^ However, there are also various factors that affect the quality of clinical decisions in clinical practice, such as team composition, standardized diagnosis and treatment mode, communication ability of members, team management, and decision-making ability,^26^ resulting in some false negative and false positive problems in MDT diagnosis.

The AI-MDT combination significantly improved diagnostic accuracy. AI's lower specificity may be attributed to its limited ability to identify rare benign lesions. In this study, cases misclassified by AI-such as inflammatory pseudotumors were corrected during MDT review based on imaging features, clinical history, and laboratory data. This demonstrates how multidisciplinary collaboration can compensate for AI limitations and reduce diagnostic error.

International evidence supports this synergistic effect. Tsakok et al.^27^ found that AI-MDT collaboration reduced the false-negative rate of small nodules from 12 % to 4 %, while Andrew et al.^28^ reported improved decision-making efficiency and reduced cognitive workload. These findings align with these results and underscore the potential utility of this combined approach.

Despite its promise, widespread adoption of AI-MDT models faces challenges. High implementation costs, data storage requirements, and the need for coordinated specialist participation may limit scalability.^29^ Resource constraints, particularly in primary care settings, further hinder MDT development.^30^ Moreover, AI generalizability depends on diverse, multicenter training datasets-currently a limitation for many models. In addition to these factors, this study has several inherent limitations that should be acknowledged. First, the relatively small sample size may restrict the statistical power and limit the robustness of the conclusions. Second, the absence of external validation using independent datasets from other institutions reduces the model’s generalizability. Third, potential selection bias may have arisen due to the retrospective study design and inclusion criteria, which might not fully represent the broader clinical population. Lastly, differences in disease prevalence, healthcare infrastructure, and diagnostic workflows across regions may further limit the applicability of these findings to other settings.

Although a multivariable logistic regression analysis or sensitivity analysis could potentially enhance the robustness of the findings, such analyses were not conducted in the present study due to the limited sample size and retrospective data structure.

Future research involving larger, prospectively collected datasets will be necessary to support more comprehensive statistical modeling and to better account for potential confounding factors. Moreover, the authors need to promote the popularization of AI-MDT model through policy support and technology optimization, as well as enhance multi-center data sharing to improve the generalization performance of AI.

## Conclusion

In this study, the combination of AI-based chest CT and MDT consultation demonstrated improved diagnostic performance in distinguishing between benign and malignant pulmonary nodules compared to either approach used alone. The integration of AI and MDT may help reduce clinical workload and support more informed diagnostic decisions.

However, the findings should be interpreted with caution. While the combined approach showed promising results, its clinical utility and stability require further prospective validation and assessment using external datasets. Therefore, additional large-scale and multicenter studies are necessary before the approach can be widely implemented in clinical practice.

## Ethical statement

This research was approved by Binzhou People's Hospital ethical committee, Approved (LL-2023) NO. 008. The authors followed the Helsinki guidelines.

## Funding

This study was supported by the Commercial research funds from Binzhou People's Hospital (Z-2020–09–2107) and Wu Jieping Medical Foundation (320.6750.2023–16–18).

## CRediT authorship contribution statement

**Xian-Yan Liu:** Data curation, Formal analysis, Validation, Visualization, Writing – original draft. **Fa-Cheng Shan:** Investigation, Methodology. **Hui Li:** Project administration. **Jian-Bo Zhu:** Funding acquisition, Resources, Software, Supervision, Writing – review & editing.

## Declaration of competing interest

The authors declare no conflicts of interest.
